# Effect of polysaccharide from *Bacillus subtilis sp*. on cardiovascular diseases and atherogenic indices in diabetic rats

**DOI:** 10.1186/s12906-016-1093-1

**Published:** 2016-03-31

**Authors:** Mona A. M. Ghoneim, Amal I. Hassan, Manal G. Mahmoud, Mohsen S. Asker

**Affiliations:** 1 Department of Radioisotopes, Nuclear Research Centre, Atomic Energy Authority, Dokki, Giza, Egypt; 2 Microbial Biotechnology Department, National Research Centre, 33 Bohouth St., Dokki, Giza, 12311 Egypt

**Keywords:** Streptozotocin, *Bacillus subtilis*, Exopolysaccharide, Diabetic, Lipid profile, Cardiovascular disease

## Abstract

**Background:**

Diabetes mellitus induces chronic complications such as cardiovascular damage, cataracts and retinopathy, nephropathy, and polyneuropathy. The main aim of the study was to isolate and identify both of bacterial strain and exopolysaccharide to assess the possible efficiency of exopolysaccharide (BSEPS) from *Bacillus subtilus sp .suppress* on cardiovascular diseases, atherogenic and coronary risk indices in diabetic rats.

**Methods:**

The bacterial strain used was isolated from mangrove tree sediment by serial dilution and the spread-plate technique and identified by morphological, physiological, and biochemical characteristics, and by 16S rRNA analysis. The BSEPS was extracted from the bacterial supernatant by four volumes child ethanol then the functional groups, MW and chemical analysis were detected by Fourier-transform infrared (FTIR), gel permeation chromatograph (GPC) and High-performance liquid chromatography (HPLC). Also an antioxidant activity was measured by using 2,2-diphenyl-1-picrylhydrazyl (DPPH). Thirty-two male Sprague–Dawley rats were equally randomized into four groups: control group supplemented with normal saline (Group I); the second group supplemented with BSEPS (Group II); diabetic group supplemented with normal saline (Group III) and the diabetic group supplemented with BSEPS (Group IV). Diabetes was induced by Streptozotocin (STZ) (65 mg/kg BW) intraperitoneally. BSEPS (100 mg/kg BW) was administered orally for four weeks, following STZ induction.

**Results:**

The isolated strain was identified based on 16S rRNA sequence as *Bacillus subtilis* sp. *suppress.* A preliminary chemical analysis of BSEPS indicated that the monosaccharides were mannuronic acid, glucuronic acid, glucose, galactose, and mannose in a molar ratio of 1.6:1.5:1.0:2.3:1.4, respectively, with a molecular weight of 1.66 × 10^4^ g mol^−1^ and a molecular number of 7.64 × 10^3^ g mol^−1^. BSEPS inhibited 2,2-diphenyl-1-picrylhydrazyl radical activity, and BSEPS supplement reduced glucose (*p <* 0.05) and troponin levels while insulin levels increased (*p <* 0.05). BSEPS also reduced total serum cholesterol, low-density lipoprotein (LDL), very low-density lipoprotein (VLDL), and triglycerides, and elevated high-density lipoprotein-cholesterol (HDL). In parallel, intercellular adhesion molecule (ICAM), and vascular cell adhesion molecule (VCAM) levels in STZ-induced diabetic rats were reduced. Moreover, polysaccharides reduced atherogenic and coronary risk indices, which were confirmed by histopathological examination of the heart and aorta.

**Conclusions:**

Our study suggests that BSEPS improves hyperglycemia, dyslipidemia, and cardiovascular disease risk in STZ-induced diabetic rats.

## Background

Diabetes mellitus is a heterogeneous combination of chronic perturbations of carbohydrate, lipid, and protein metabolism characterized by increased blood glucose owing to partial or absolute insulin deficiency [1]. The World Health Organization estimates that diabetes affects 285 million people globally, and current predictions estimate that 438 million people will be diabetic by 2030 [2, 3]. Pharmacological treatment of diabetes relies on oral hypoglycemic agents and insulin, but these approaches currently used in clinical practice either do not restore normal glycemic levels in most patients or their levels change over time. Moreover, continuous use of synthetic anti-diabetic drugs causes side effects and toxicity [4]. Because of the limitations and unmet goals associated with anti-diabetic drugs, an increased number of diabetic patients globally currently resort to complementary and alternative medicine [5]. Therefore, natural and non-toxic anti-diabetic drugs are inevitable for diabetic therapeutics such as exopolysaccharides (EPSs) [6]. Metabolic abnormalities and oxidizing stress arise at early stages of diabetes and are significant risk factors for complications of the nervous, cardiovascular, excretory, and reproductive systems [7]. Several remedial agents are effective for hyper-cholesterolemic patients and are used successfully for treatment. A variety of studies have demonstrated that the mechanism of lipid-lowering drugs can reduce the number of cardiovascular events and mortality from coronary disease [8]. EPSs are natural macromolecules present throughout the growth of many microorganisms. An inordinate number of bacterial EPSs has attracted the interest of many scientists who have examined their composition, structure, biosynthesis, and functional properties [9]. A number of reports have been published on the anti-atherosclerosis effect of acidic EPSs and the inhibition of total cholesterol [10]. Atherosclerosis is combined with a broad spectrum of cardio-metabolic disturbances, including diabetes, arteriosclerosis, and cardiovascular disease. *Bacillus* sp. is one of the marine bacteria that produce complex homo-and/or heteropolysaccharides. *Bacillus* sp. produce a variety of EPSs such as levan [11] and β-(1–3)-glucan [12], and hetero-polymers composed mainly of neutral sugar [13], uronic acid [14], uncommon sugar [15], or sugar protein conjugates [16]. The form and mechanisms of the pharmaceutical effects of medical specialty EPSs on diseases have been largely elucidated, and normal EPSs with different curative potential have been studied and even included in therapies. Thus, the present study was designed to evaluate the potential beneficial effect of the exopolysaccharides (BSEPS) from *Bacillus subtilus sp. suppress* from marine on serum lipid parameters and cardiovascular disease risk predictor in normal and Streptozotocin-induced diabetic rats. Additionally, the chemical structure of purified BSEPS was elucidated after identification of isolated bacterial strain.

## Methods

### I- In vitro study

#### Screening and identification

Marine sediment collected from the mangrove tree (El-Ein El-Sokhna, Egypt) was suspended in 90 mL sterile water. Serial dilution and the spread-plate technique were used for growing different bacteria. The screening medium contained (all reagents in g/L) glucose 20; yeast extract 0.1; CaCO_3_ 1; NH_4_NO_3_ 0.8; K_2_HPO_4_ 0.6; KH_2_PO_4_ 0.5; MgSO_4_.7H_2_O 0.05, and MnSO_4_.4H_2_O 0.1 in 75 % sea water [17]. The initial screening selected for smooth, ropy, and mucous colonies from each plate, with subcultures maintained on marine nutrient agar medium until a pure isolate was obtained. Pure colonies were then inoculated into 50 mL of marine nutrient medium in a 250-mL conical flask, and incubated at 37 °C in a rotary shaker at 120 rpm for 48 h. After centrifugation, supernatant was mixed with four volumes of chilled ethanol. The precipitate was collected by centrifugation and the pellets washed by acetone and diethyl ether, and dried at 50 °C until constant weight. EPS production was determined by the phenol-sulfuric acid method [18]. Strain NRC-108, which produces elevated EPS levels, was identified on the basis of morphological, physiological, and biochemical characteristics [19] combined with 16S rRNA sequence analysis. The universal primers delineated by Weisburg et al. [20], particularly ITS1 and ITS4, were used to amplify the 16S rRNA gene sequence. A single, discrete, polymerase chain reaction amplicon was resolved on agarose. Sequencing products were resolved on an Applied Biosystems (Foster City, CA, USA) model 3730 XL, automated DNA sequencing system. Data were submitted to GenBank and the sequence compared with the GenBank database (http://www.ncbi.nlm.nih.gov) using BLAST [21].

### Isolation and purification of EPSs

Inoculums was prepared by transferring one loop full of culture (NRC-108) from marine nutrient slant to an Erlenmeyer flask (250 mL) consisted of 50 ml seed medium, (g/L) sucrose 20; yeast extract 2; peptone and 75 % sea water [22]. The seed cultures were grown at 37 °C on a rotary shaker incubator at 150 rpm for 24 h. After incubation, 3 ml of the seed culture was transferred into an Erlenmeyer flask (250-mL) containing 50 ml of fermentation medium (g/L) sucrose 50; peptone 4; yeast extract 2 in 75 % sea water pH 7.0 [23]. The fermentation cultures were then incubated at 37 °C with shaking at 150 rpm for 3 days. The fermented broth was collected and centrifuged at 3500 xg at 4 °C for 30 min and dialyzed three times (1000 mL × 3) against flowing tap-water using dialysis tubing (MWCO 2000) for 48 h. The dialyzed solution through precipitation with four volumes chilled ethanol, the precipitate washed with acetone, diethyl ether and dried at 40 °C until constant weight. The crude EPS was re-dissolved in deionizaed water and forced through a filter (0.45 mm), then applied to a column (2.5 × 70 cm, i.d.) of DEAE-cellulose. After loading with a sample, the column was eluted with a gradient NaCl solution (0.0–1.0 M), and the procedure was monitored by the phenol-H_2_SO_4_ method mentioned above. One polysaccharide active fraction (BSEPS) was collected, dialyzed and lyophilized. BSEPS was used for activity assessment and structural analysis [24].

### Chemical analyses

Purified BSEPS (50 mg) was subjected to hydrolysis with 6 N HCl for 4 h at 100 °C in a sealed tube. Excess acid was removed by evaporation on a water bath at 40 °C and co-distilled with water (1 mL × 3) [25]. Uronic acid content was determined by measuring the absorbance at 525 nm using the m-hydroxybiphenyl colorimetric procedure and with glucuronic acid as the standard [26]. Sulfate was measured using the turbidimetric method [27] with sodium sulfate as the standard. N-acetylglucosamine was estimated by the Morgan and Elson reaction [28]. Ultraviolet–visible (UV–vis) spectroscopy analyses were conducted on a UV–vis spectrophotometer (2401PC; Shimadzu, Kyoto, Japan). BSEPS solution was prepared by suspending the sample in distilled water to 1.0 mg/mL for UV–vis measurement at 190–700 nm. BSEPS monosaccharide composition was determined by High Performance Liquid Chromatography (HPLC) on a Shimadzu Shim-Pack SCR-101 N column (7.9 mm × 30 cm, i.d.), using deionized water as the mobile phase (flow rate 0.5 mL/min), as described by El-Sayed et al. [29].

### Fourier-transform infrared spectroscopy

The Fourier-transform infrared spectrum of BSEPS was measured on a Bruker 500-IR Spectrophotometer (Billerica, MA, USA). The sample was mixed with KBr powder, ground, and pressed into 1-mm pellets for Fourier-transform infrared measurements in the range of 400–4000 cm^−1^ [30].

### Molecular-weight determination

BSEPS molecular weight was determined on an Agilent 1100 HPLC system equipped with a refractive index detector and FPl gel particle (5 μm), three columns of pore type (100, 104, and 105 A°) in series, length 7.5 × 300 mm (1000, 5000000) for DMF solvent Styrogel HR-DMF, 3 μm (7.8 × 300 mm) (Waters, Milford, MA, USA). One column (5000–600,000) was used for water solvent (polyethylene oxide/glycol standard) PL aquagel, OH 7.5 mm and 30 μm pore, 8 um particle size. Sample (0.01 g) was dissolved in 2 mL of solvent and filtrated (0.45 mm) and transferred to a gel-permeation chromatography (GPC) device [31]. Number average molecular weight (Mn) and number average molecular weight (Mw) were directly calculated according to the definition of Mn and Mw using molecular weight and refractive index signal values at each elution volume [32].

### BSEPS scavenging activity of DPPH free radicals

BSEPS free radical scavenging activity was measured against DPPH radicals using the method of Asker et al. [33]. DPPH ethanol solution (5 mL, freshly prepared) was added to 1 mL of BSEPS solution (30, 50, 70, and 100 μg mL^−1^). Solutions were mixed vigorously and incubated at room temperature in the dark for 30, 60, 90, and 120 min. Supernatant absorbance was measured at 517 nm. A lower absorbance indicates higher free radical scavenging activity, as determined from graphing inhibition percentage plotted against compound concentration. All experiments were carried out in triplicate and averaged. The ability to scavenge DPPH radicals was calculated using the following equation:

$$ \mathrm{Scavenging}\ \mathrm{ability}\ \left(\%\right) = \left[\left({\mathrm{OD}}_{517\mathrm{of}\ \mathrm{control}}\hbox{--}\ {\mathrm{OD}}_{517\mathrm{of}\ \mathrm{sample}}\right)/{\mathrm{OD}}_{517\mathrm{of}\ \mathrm{control}}\right] \times 100 $$

### II- In vivo study

#### Animals

Thirty-two male Sprague–Dawley rats (180–200 g) were used in this study. Rats were purchased from the laboratory animal colony of the Egyptian Organization for Biological Products and Vaccines (VACSERA) (Helwan, Cairo, Egypt). The animals were housed in the Animal House of the Radioisotopes Department, Atomic Energy Authority, Egypt, under hygienic conditions and fed for 1 week on a basal diet for adaptation and supplied with water ad libitum. The Animal Care Committee of the National Centre for Radiation Research and Technology, Cairo, Egypt, approved the treatment protocol, following the guidelines of the National Institutes of Health.

### Induction of diabetes in experimental rats

Diabetes was induced by a single intraperitoneal injection of a freshly buffered (0.02 M citrate, pH 4.5) solution of STZ (Sigma Chemical Co., St. Louis, MO, USA) at a dosage of 65 mg/kg body weight, as described previously [34]. After 72 h of STZ administration, tail vein blood was collected to determine fasting blood glucose levels. Rats with fasting blood glucose ≥200 mg/dL were considered diabetic and included in the experiments [35]. Treatment with EPS was after the last STZ injection. Blood samples were drawn at the end of the study (30 days).

### Acute toxicity (LD_50_) test for BSEPS

Acute toxicity (LD_50_) was determined for 18 rats in two phases using the method of Lorke [36]. In the initial phase, the rats were divided into three groups of three rats each. They were orally treated with 10, 100, and 1000 mg/kg of extract and observed for 24 h for mortality. In the second phase, rats were grouped into three groups of three rats each and orally treated with the extract at varying doses (1600, 2900, and 5000 mg/kg). Rats were observed for 24 h and the final LD_50_ value was determined from the minimum concentration (death) and maximum concentration (survival) of the dose as follows:

$$ \mathrm{L}{\mathrm{D}}_{50} = \left({\mathrm{M}}_0 + {\mathrm{M}}_1\right)/2, $$

where M_0_ is the highest dose of test substance with survival and M_1_ is the lowest dose of test substance resulting in death.

### Experimental design

After 1 week of adaptation, rats were divided into four groups of eight rats each as follows: Group I served as a negative control (a single intraperitoneal injection of freshly buffered [0.02 M citrate, pH 4.5] without STZ) [34]. Rats were injected with normal saline (0.5 mL, orally) 3 days later and every other day for 30 days. Group II was administered normal BSEPS (100 mg/kg BW) dissolved in saline. Group III was given STZ (65 mg/kg BW) dissolved in citrate buffer (pH 4.5) and injected intraperitoneally within 10 min after preparation. Rats were administered with normal saline (0.5 mL, orally) 3 days later and every other day for 30 days. Group IV consisted of diabetic rats who received daily BSEPS orally, for 30 days. Normal groups were provided common commercial rat chow. Diabetes was confirmed after 72 h of STZ injection. Blood samples were collected through the caudal vein and blood glucose levels estimated by diagnostic kits. Rats having blood glucose levels more than or equal to 200 mg/dL were selected and used for the study.

### Biochemical analysis

At the end of the experiment animals were fasted overnight, anesthetized, and sacrificed to obtain blood samples. Each blood sample was placed in a dry clean centrifuge tube, and centrifuged for 10 min at 2100 xg to separate the serum. Serum was carefully separated into clean dry Wassermann tubes using a Pasteur pipette and kept at −80 °C until analysis.

Serum glucose level (mg/dL) was determined using a glucose test kit (Egy-Chem, Cairo, Egypt) based on the Trinder et al. glucose oxidase method [37]. Insulin levels were determined by radioimmunoassay according to Clark et al. [38]. Troponin T levels were determined by means of a third-generation troponin T assay (Elecsys; Roche Diagnostics, Indianapolis, IN, USA) that uses recombinant cardiac troponin T as a standard [39]. Triglyceride was determined by the method suggested by Trinder et al. [40]. Total cholesterol was estimated by the method described by Roeschlau et al. [41]. HDL-C and LDL-C were measured using the method described by Assman et al. [42] and VLDL-C was measured using the formula Triglyceride/5 = VLDL-C (mg/100 mL).

### Atherogenic index

The atherogenic index (AI) was determined by the formula suggested by Haglund et al. [43]. Briefly, AI = (TC-HDL)/HDL and coronary heart index (CRI) was calculated as described by Ishiguro et al. using the formula: CRI = Total cholesterol/HDL-cholesterol [44].

### Molecular markers of atherosclerosis and coronary heart disease

VCAM-1 and ICAM-1 serve as molecular markers of atherosclerosis and predictors of incident coronary heart disease, respectively. ICAM-1 and VCAM-1 levels were determined using Duo Set ELISA kits (R&D Systems, Minneapolis, MN, USA), in 96-well plates following the manufacturer’s protocol. Absorbance was measured at 450 ± 10 nm with a Stat Fax 2100 Microplate Reader (MidSci, St. Louis, MO, USA). Levels of VCAM-1 and ICAM-1 were normalized using the total cellular protein concentration, which is proportional to cell number.

### Histological examination

Autopsy samples were taken from the aorta and fixed in 10 % formal saline for 24 h, washed in tap water, then dehydrated in serial dilutions of alcohol (methyl, ethyl, and absolute ethyl). Specimens were cleared in xylene and embedded in paraffin at 56 °C in a hot air oven for 24 h. Paraffin bees wax tissue blocks were prepared for sectioning at 4 microns (μm) thickness by Sledge Microtome (Leica Biosystems Inc., Buffalo Grove, IL, USA). Tissue sections were collected on glass slides, deparaffinized, and stained by hematoxylin and eosin stain for examination by light microscopy [45].

### Statistical analysis

Data analysis was carried out with SPSS Inc. software V22 (IBM, Chicago, IL, USA). One-way analysis of variance was used to determine statistically significant differences between dietary groups (*P* < 0.05). Duncan’s test was used to compare the significance among groups. All data are presented as ± standard error of the mean.

## Results

### Screening and strain identification

**Fig. 1:**
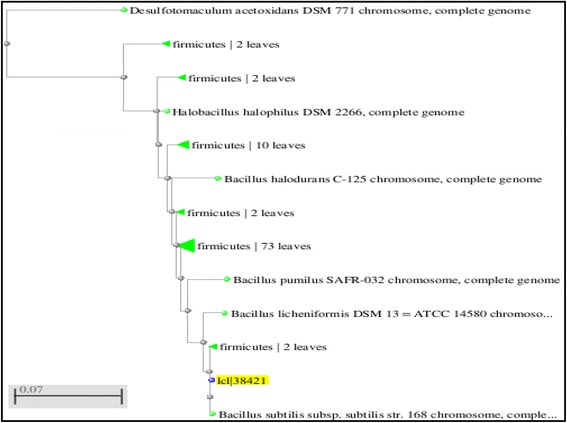
Phylogenetic neighbor joining tree obtained with the 16S rDNA sequences of strain NRC-108 and members of related bacteria

EPSs from different strains may be different in yield, chemical composition, structure, and physical properties. Forty-two bacterial isolates were isolated from soil samples, with 19 isolates obtained based on the carbohydrate content of their supernatant broth. Although most isolates produced EPS, strain NRC-108 was selected for further study because it demonstrated the highest EPS production (9 g/L). NRC-108 colonies exhibited a creamy mucous on solid medium, and were Gram-positive, rod shaped, and spore forming. The strain produced amylase and arginine and was negative for nitrate reduction, positive for Voges-Proskauer and indol tests, and acid production. Hence, strain NRC-108 was tentatively identified as *Bacillus* sp. Comparison of 16S rRNA gene sequence among NRC-108 and other bacteria from GenBank by BLAST analysis showed the closest strains (99 % similarity) were *B. subtilis*, *B. licheniformis* DSM13, and *B. pumilus* SAFR-032. Phylogenetic analysis results indicated that NRC-108 was most closely related to *B. subtilis* (Fig. 1). The strain is therefore identified as *B. subtilus sp. suppress*.

### Production and isolation of BSEPS

**Fig. 2:**
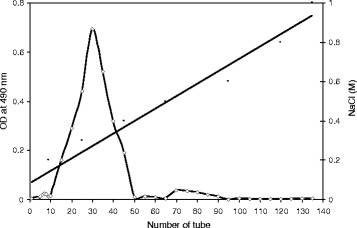
Elution curve of BSEPS from *Bacillus subtilis* sp. over DEAE-cellulose column. The absorbance at 490 nm was that of the resulting reactive solutions of exopolysaccharides, phenol and sulfuric acid

NRC-108 produced high levels of EPS (10.5 g/L) when fermented at 35 °C for 96 h, as determined by the phenol–sulfuric acid assay using glucose as a standard. Crude EPS was obtained from fermented broth by ethanol precipitation and dehydration by acetone and diethyl ether. The crude EPS was fractionated on a DEAE-cellulose anion exchange chromatography column (Fig. 2). One major EPS peak was eluted with 0.3 M NaCl, concentrated, dialyzed, and lyophilized to obtain purified BSEPS (white-yellow powder), which was used for subsequent analyses. It produced a positive response to the Bradford test and absorption at 280/260 nm, indicating the presence of protein and/or nucleic acid. UV–vis spectroscopy analysis showed that the maximum absorption was 200–220 nm owing to n–σ* and/or π-π* transitions, characteristic of amine functional groups.

**Fig. 3:**
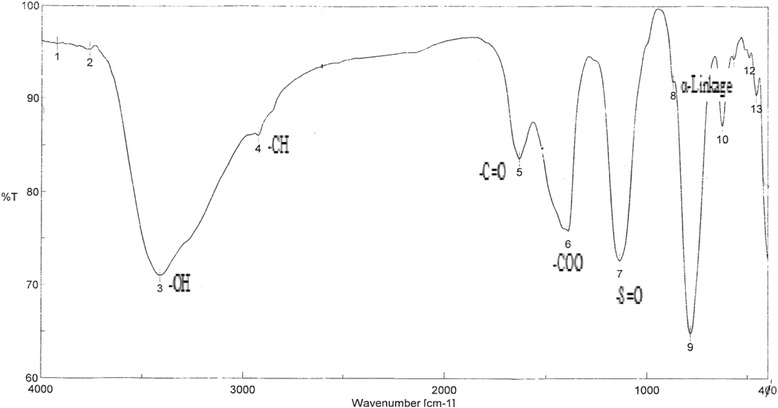
IR spectrum of the BSEPS from *Bacillus subtilis* sp. in the range 400–4000 cm^−1^

The largest absorbance peak at 210 nm is characteristic of BSEPS. There was weekly absorption at 280 nm, indicating very small amounts of protein. BSEPS also contained 35.4 % uronic acid as evaluated by m-hydroxydiphenyl colorimetry, 4.9 % sulfate, and 4.1 % N-acetyl glucose amine. The BSEPS FT-IR spectrum (Fig. 3) displayed a strong band at 3410.49 cm^−1^, attributed to polysaccharide O-H stretching vibration. The 2927.41 cm^−1^ band was due to C–H stretching vibration. A symmetrical stretched peak near 1388.50 cm^−1^ indicated the presence of carboxyl groups. The prominent absorption at 1633.41 cm^−1^ was attributed to C = O and C-N stretching vibrations, whereas absorption at 1132.97 cm^−1^ was due to the presence of S = O and/or C–O–S bonds. Furthermore, characteristic absorptions at 869.74 cm^−1^ suggest that α-configurations were simultaneously present in BSEPS [46].

**Fig. 4:**
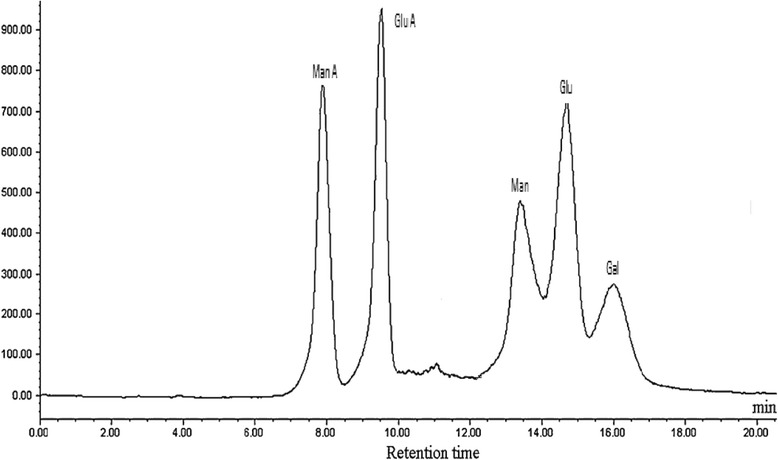
HPLC chromatogram profile of BSEPS hydrolysate

**Fig. 5:**
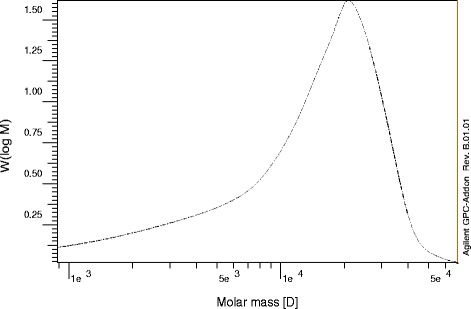
Weight average molecular weight (Mw) and number average molecular weight (Mn) distributions of BSEPS production by *Bacillus subtilis* sp

The BSEPS chemical structure was analyzed by HPLC and compared with monosaccharide standards. BSEPS was composed of mannuronic acid, glucuronic acid, glucose, galactose, and mannose in a molar ratio of 1.6:1.5:1.0:2.3:1.4, respectively (Fig. 4). EPSs from marine bacteria are primarily heteropolysaccharides consisting of pentoses, hexoses, amino sugars, or uronic acids arranged in repeating units. In order to the chemical composition determines position; so, the EPSs contain galactose and mannose that form an unstirred water layer in the gut, which decreases absorption of sugars and lipids. This effect is a property of EPS structure that, by decreasing the rate of gastric emptying, and resisting the convective effects of intestinal contractions, decreases sugar absorption by the small intestine. Thus, to some extent, soluble fibers can be used to prevent the postprandial increase of glucose, making it useful for the treatment of diabetes [47]. EPSs from marine bacteria usually possess hydroxyl and carboxyl groups, which confer a net negative charge and acidic properties. BSEPS molecular weight was calculated for the portions of peaks that lie within the peak ranges. BSEPS M*w* was determined to be 1.66 × 10^4^ g mol^−1^ and the M*n* to be 7.64 × 10^3^ g mol^−1^. The polydispersity index (Mw/Mn) is a measure of the width of molecular-weight distribution 2.17 (Fig. 5).

Most marine EPSs are linear overall and of varying lengths, with a mean molecular mass of 1 × 10^5^–3 × 10^5^ Da. Organic or inorganic substitutes may also be present [48]. In recent years, there have been reports of deep sea hydrothermal bacteria secreting EPSs; they are typically acidic and contain uronic acid (10–40 %) and a large molecular weight of up to 1 × 10^6^ g mol^−1^ [49]. The hypotriglyceridemic effect might be owing to a delayed absorption of triglyceride in the small intestine caused by the high viscosity of the intestinal contents [50]. Therefore, the viscous structure and molecular weight of polysaccharides are thought to be important for limiting absorption in the digestive tract, and increasing cholesterol excretion [50].

### BSEPS scavenging activity of DPPH free radicals

BSEPS was found to significantly inhibit the activity of DPPH radicals in a dose-dependent manner at concentrations from 30 to 100 μg/mL [32, 33]. Additional BSEPS (100 μg/mL) lead to increased viscosity and turbidity, prompting us to stop treatment.

Serum glucose and troponin levels increased (*p* < 0.05), and serum insulin levels decreased (*p* < 0.05) in STZ-induced diabetic rats compared with controls (Table 1). EPS supplementation reduced serum glucose and troponin T levels and increased insulin levels (*p* < 0.05) in the STZ-induced diabetic rats (Group IV).

**Table 1:** Effect of treatment with polysaccharide on glucose, insulin and troponin concentrations in control, and STZ-induced diabetic rats

Groups	Group I	Group II	Group III	Group IV	F	*P*-value
Glucose (mg/dl)	78.48 ± 7.37^b^	83.60 ± 2.99^b^	206.50 ± 6.24^a^	82.70 ± 3.08^b^	140*	0.000
Insulin (μIU/mL)	31.38 ± 1.24^a^	30.47 ± 2.33^a^	11.56 ± 1.67^b^	27.34 ± 0.99^a^	31.87*	0.000
Troponin-T(pg/mL)	31.43 ± 3.14^b^	33.50 ± 2.53^b^	47.27 ± 2.18^a^	34.65 ± 2.59^b^	7.40*	0.005

Data expressed as mean ± SE

^a,b^The groups in the same raw with different letters are statistically significant (*P < 0.05) using one-way ANOVA followed by Duncan as a post-hoc test

### Serum lipid profile

The present results noted an increase in serum total cholesterol, triglyceride, LDL-cholesterol, and VLDL-cholesterol levels in STZ-treated rats (*p* < 0.05) and decreased HDL-cholesterol levels compared with normal rats (*p* < 0.05) (Table 2). Interestingly, BSEPS treatment of STZ rats decreased serum lipid profiles (total cholesterol, LDL-cholesterol, VLDL-cholesterol, and triglycerides) (*p* < 0.05) and increased HDL (*p* < 0.05).

**Table 2:** Effect of treatment with polysaccharide on lipid profile in control, and STZ-induced diabetic rats

Groups	Group I	Group II	Group III	Group IV	F	*P*-value
Total cholesterol (mg/dl)	43.70 ± 1.53^b^	44.52 ± 1.46^b^	101.93 ± 7.38^a^	48.20 ± 2.46^b^	45.86*	0.000
Triglycerides (mg/dl)	40.75 ± 1.72^b^	45.43 ± 1.59^b^	130.82 ± 3.23^a^	47.22 ± 2.73^b^	309.01*	0.000
LDL-C (mg/dl)	11.48 ± 3.29^b^	8.80 ± 3.24^b^	64.33 ± 7.26^a^	7.27 ± 1.91^b^	38.54*	0.000
HDL-C (mg/dl)	28.10 ± 1.92^a^	27.58 ± 2.47^a^	11.43 ± 0.51^b^	32.21 ± 1.46^a^	29.29*	0.000
VLDL-C (mg/dl)	8.15 ± 0.34^b^	9.09 ± 0.32^b^	26.16 ± 0.65^a^	9.44 ± 0.55^b^	309.01*	0.000

Data expressed as mean ± SE

^a,b^The groups in the same raw with different letters are statistically significant (*P < 0.05) using one-way ANOVA followed by Duncan as a post-hoc test

### AI, CRI, VCAM, and ICAM

STZ control rats exhibited a profound increase in AI compared with normal rats (Table 3), suggesting that BSEPS treatment decreases AI. Similarly, VCAM and ICAM levels increased in the diabetic group compared with controls, while STZ-induced diabetic rats treated with EPS typically improved, with parameters returning to control levels. Similarly, the data indicate that STZ increases the CRI compared with normal rats (8.90 ± 0.56 vs. 1.6 ± 0.19). In addition, BSEPS-treated rats exhibited an improved CRI (1.51 ± 0.09), compared with diabetic rats. The acute toxicity data indicate the LD_50_ was 600 mg/kg, which implies that at this concentration, 50 % of the animals would be killed (Table 4).

**Table 3:** Effect of treatment with polysaccharide on serum concentration of ICAM and VCAM in control, and STZ-induced diabetic rats

Groups	Group I	Group II	Group III	Group IV	F	*P*-value
AI (mg/dl)	0.59 ± 0.17^b^	0.68 ± 0.2^b^	7.73 ± 0.59^a^	0.51 ± 0.08^b^	112.97*	0.000
CRI (mg/dl)	1.60 ± 0.19^b^	1.58 ± 0.20^b^	8.90 ± 0.56^a^	1.51 ± 0.09^b^	128.204*	0.000
ICAM (ng/ml)	6.03 ± 0.12^c^	5.21 ± 0.13^d^	7.96 ± 0.25^a^	6.89 ± 0.17^b^	42.00*	0.000
VCAM (ng/ml)	16.82 ± 1.34^b^	17.35 ± 0.69^b^	23.98 ± 0.45^a^	18.34 ± 0.99^b^	27.90*	0.000

Data expressed as mean ± SE

^a,b,c,d^The groups in the same raw with different letters are statistically significant (*P < 0.05) using one-way ANOVA followed by Duncan as a post-hoc test

**Table 4:** Pharmacological Study Acute Toxicity (LD) Testing of BSEPS

Doses (mg/kg)	Result of first phase (mortality) *n* = 3 in each group
10	0/3
100	0/3
1000	1/3
Doses (mg/kg)	Result of 2^nd^ phase (mortality) *n* = 3
1600	1/3
2900	2/3
5000	2/3

The LD50 value of BSEPS = 600 mg/kg

### Histopathological findings

**Fig. 6:**
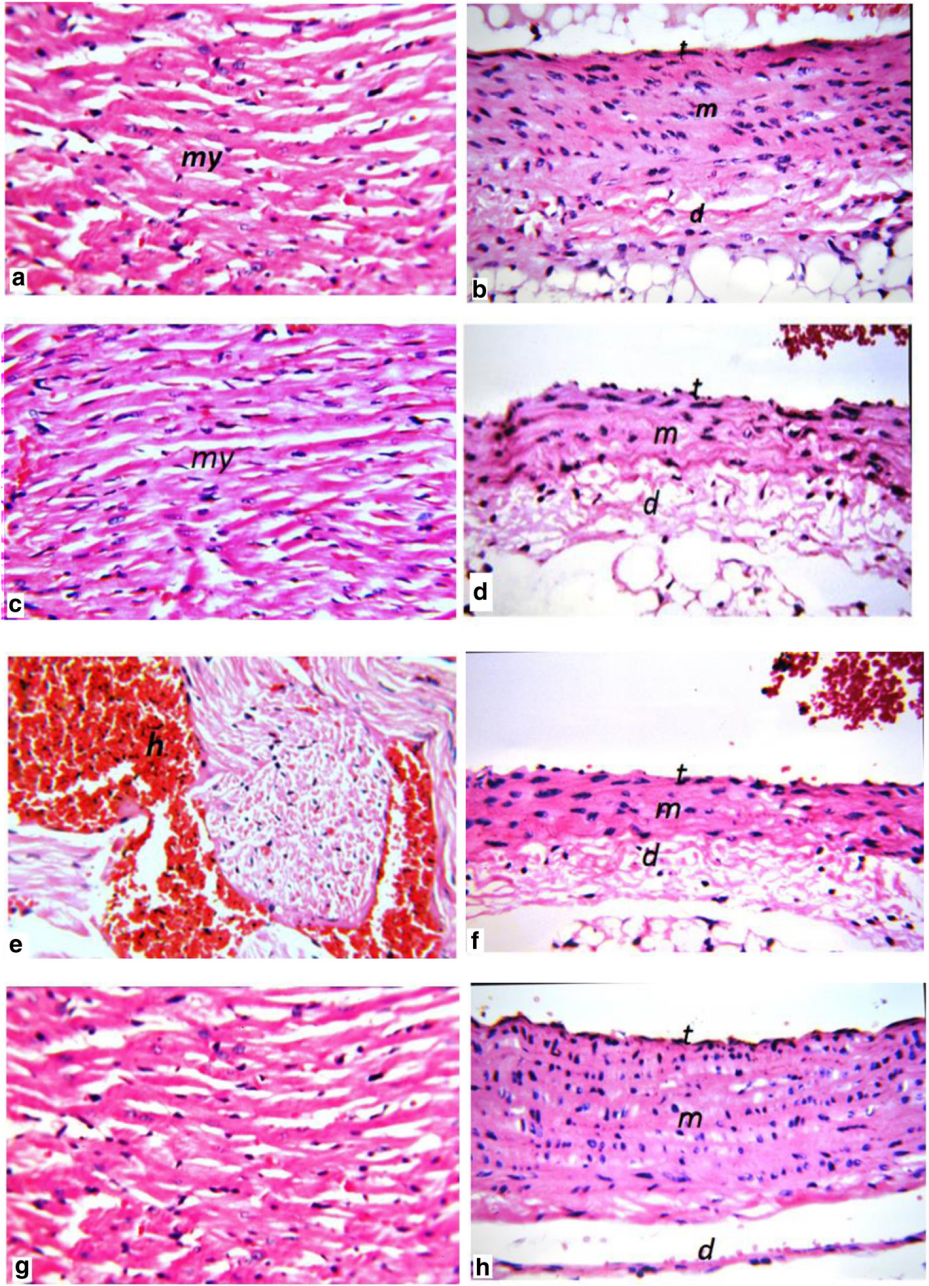
Heart: (**a**). Group I There was no histopathological alteration and the normal histological structure of the myocardium H& E × 64. Aorta: (**b**) Group I There was no histopathological alteration and the normal histological structure of the tunica intima, media and adventitia H& E × 64. **c**: Group II There was no histopathological alteration as recorded in heart tissue H& E × 64. Aorta (**d**): Group II There was no histopathological alteration as recorded H& E × 64. **e**: Group III, Focal haemorrhages were detected in the myocardium H& E × 64. Aorta (**f**): There was oedema in the adventitia (group III) H& E × 64. **g**; Group IV, there was no histopathological alteration the normal histological structure of the myocardium H& E × 64. Aorta (**h**): Group IV there was no histopathological alteration as recorded H& E × 64

We did not observe any major histological differences between groups GI and GII in either the heart tissues or the aorta, by hematoxylin and eosin staining (Fig. 6a–d). In STZ rats beginning on the 4^th^ week, focal hemorrhages, congestion, perivascular edema in the small intramuscular arterioles, and focal muscular hyalinization were consistently observed in the heart (Fig. 6e). The focal area in the degenerated myocardium displayed myo-fibroblast proliferation with a little infiltration of inflammatory cells, as well as congestion in the blood vessels of GIII rats (Fig. 6e). There was also edema in the adventitia, and the aortic tunica intima was irregular. In addition, smooth muscle cells with a perinuclear halo were seen in the tunica media. In contrast, BSEPS-treated STZ rats did not show any appreciable change in the heart (Fig. 6f). Normal small intramyocardial arteries were seen in the single-cell layer endothelium. No obvious damages of the aorta were observed in the BSEPS-treated group IV (Fig. 6g).

## Discussion

The understanding of cardiac abnormalities that accompany diabetes is of high clinical relevance because, curiously, diabetes increases the risk of coronary -artery disease [51]. Hydroxyl radicals are the most reactive of all the reduced forms of dioxygen and are believed to initiate cell damage both in vitro and in vivo [52]. Gille et al. [53] conclude that STZ-mediated hydroxyl radicals and the generation of reactive oxygen species may be crucial effectors in β-cell damage. Our data show a significant decrease in the concentration of DPPH radicals owing to the scavenging ability of BSEPS.

Our study investigated the effect of EPS treatment on the lipid profile and cardiovascular disease risk predictor (AI and CRI) in STZ-induced diabetic rats. Generally, increase blood glucose levels characterize STZ-induced diabetes, consistent with our results. Previously, STZ diabetic animals were shown to exhibit tissue injury and diabetic complications that involve the cardiovascular, gastrointestinal, and nervous systems as well as the vas deferens, kidney, eyes, and urinary bladder through increased lipid peroxidation and oxidative stress [54]. Our results indicate that diabetes induced a significant elevation of troponin levels. Diabetes triggers ongoing myocardial damage, which manifests as elevated serum cardiac troponin T, increasing the risk for further cardiac events in patients with chronic heart failure [55, 56].

Our data indicate that BSEPS accentuates hyperlipidemia as efficiently as other polysaccharides [56]. BSEPS protects against increases in glucose and decreases in insulin that might be attributed to its ability to reduce hyperglycemia. This might be via the inhibition of hepatic gluconeogenesis and glucose production from the liver, which is accompanied by suppression of lipolysis in adipose [57]. The abnormal high level of serum lipids in diabetes is mainly due to an increase in the mobilization of free fatty acids from peripheral fat depots by lipolysis [58]. Increased release of free fatty acids increases the production of ketone bodies and triglyceride synthesis. In the present investigation, triglycerides were increased significantly in diabetic control rats. Insulin deficiency depletes the activity level of lipoprotein lipase, thus leading to abnormal lipoprotein metabolism in diabetes and resulting in hypertriglyceridemia [59].

A major reason for cardiovascular disease in diabetes is the lipid abnormalities that accompany atherosclerosis. Therefore, an ideal treatment for diabetes, in addition to glycemic control, ought to facilitate production of healthy lipid profiles [60]. The present results elucidated a significant increase in serum total lipids, total cholesterol, triglyceride, LDL-cholesterol, and VLDL-cholesterol levels in STZ control rats and decreased HDL-cholesterol levels compared with normal rats. These results are consistent with those of other investigators [61]. Additionally, increased levels of serum triglycerides could also contribute to reduced clearance of triglycerides, secondary to decreased activity of lipoprotein lipase [62]. The high level of LDL-cholesterol found in STZ rats may be attributed to cholesterol-mediated down-regulation of LDL receptors [63]. Reduction in HDL following STZ may be because of the acceleration of apoA-I clearance from the plasma owing to elevated cholesterol [63]. Moreover, diabetes often presents with impaired carbohydrate metabolism, and increased lipolysis causes accumulation of acetyl CoA. Increased availability of acetyl CoA leads to synthesis of cholesterol and causes hyperlipidemia [64]. Notably, increased total cholesterol levels were restored in BSEPS-treated diabetic rats. Insulin deficiency is associated with hypercholesterolemia owing to metabolic abnormalities [65]. For example, serum lipids are increased by lipolysis as a result of insulin deficiency in diabetic rats [66]. Usually, insulin increases lipogenesis and decreases lipolysis and ketogenesis. However, in the diabetic condition, insulin deficiency reverses the above-mentioned role in lipid metabolism. Triglycerides are neutral fats and a major energy reserve for the body stored as adipose tissue. In the present investigation, triglycerides increased significantly following STZ treatment. Diabetes increases lipolysis and produces more free fatty acids. Increased free fatty acids increases production of ketone bodies and triglyceride synthesis. The increased level of triglycerides was maintained in diabetic rats following BSEPS treatment.

LDL plays a significant role in atherosclerosis and related diseases. In the present study, LDL levels increased in STZ-induced diabetic rats. Importantly, LDL transports cholesterol from the liver to peripheral tissues, and is formed from VLDL-cholesterol. VLDL levels also increased in the present study after STZ injection, denoting increased production of LDL-cholesterol. The effective control of glycemic imbalance will reduce the VLDL and triglyceride levels [67]. HDL cholesterol plays a role in preventing atherosclerosis by transporting cholesterol from peripheral tissues to the liver for excretion. A decrease in HDL was observed in the present study, which increases the chances of atherosclerosis. Restoration of the HDL decrease in treated diabetic rats shows the potential alleviating capacity of BSEPS. Increased serum total cholesterol and LDL levels are negatively correlated with HDL levels [67]. An increase in HDL-cholesterol is associated with a decrease in coronary risk, which is associated with elevated levels of total cholesterol, especially LDL-cholesterol [68]. The antihyperglycemic effect of BSEPS and hence an improved diabetic state may lead to a reduction in VLDL levels, and consequently LDL levels.

The AI and CRI are also quite high in STZ-induced diabetic rats. Elevated serum lipids often indicate coronary heart disease. BSEPS treatment improved the worst condition to its normal level as compared with the STZ group, as shown through increased HDL-cholesterol. Importantly, HDLs protect against or reverse atherosclerosis by their ability to serve as acceptor particles for macrophage cholesterol efflux, prevention of endothelial dysfunction, and maintenance of endothelial integrity [69]. Moreover, the increase in HDL cholesterol is associated with a decrease in coronary risk [70].

The histological findings showed that cardiac structural organization was disturbed in STZ-induced diabetic rats. Inflammatory histological change in cardiac tissue indicates myocardial injury. Previous studies reported diabetic cardiomyopathy as characterized by systolic or diastolic dysfunction and cardiac fibrosis in diabetes patients [71]. In a state of insulin deficiency (type 1 diabetes), there is a decrease in protein synthesis, an increase in protein degradation, and defective mitochondrial function with loss of myofibrils in the heart muscle [72, 73], which may disrupt the cardiac *myo*-fibers. Aortic wall histology of diabetic rats indicated an increase in thickness. In summary, our findings suggest BSEPS extract provides a protective role for atherosclerosis in STZ-induced diabetic rats.

## Conclusion

It is concluded that the BSEPS from *Bacillus subtilus sp. Suppress* is effective in the management of the hyperglycemias as well as dyslipidemia in induced conditions like diabetes. Treatment with BSEPS maintained the histological integrity of the cardiac tissues by reducing the degenerative changes in the myocardium as well as in the aortic tissues by decreasing the thickness of tunica media. Also free radical scavenging activity also increased with increasing concentration in the range of 30–100 μg/mL. These *in-vitro* assays of antioxidant indicate that this BSEPS is a significant source of natural antioxidant, which might be helpful in preventing the progress of various oxidative stresses.

## Abbreviations

AI: atherogenic index
CAM: complementary and alternative medicine
CDV: cardiovascular disease
CHD: coronary heart disease
DM: diabetes mellitus
DPPH: diphenyl-2-picryl-hydrazyl
EPS: exopolysaccharides
FTIR: fourier-transform infrared
GPC: gel-permeation chromatography
HCl: hydrochloric acid
HDL: high density lipoprotein
HPLC: high performance liquid chromatography
ICAM: intercellular adhesion molecule-1
KBr: potassium bromide
LDL: low-density lipoprotein
PCR: polymerase chain reaction
RID: refractive index detector
rRNA: ribosomal ribonucleic acid
STZ: streptozotocin
UV: ultraviolet
VCAM: vascular cell adhesion molecule-1
VLDL: very low density lipoprotein
WHO: World Health Organization
